# The frequency of Duchenne muscular dystrophy/Becker muscular dystrophy and Pompe disease in children with isolated transaminase elevation: results from the observational VICTORIA study

**DOI:** 10.3389/fped.2023.1272177

**Published:** 2023-09-25

**Authors:** Aydan Kansu, Zarife Kuloglu, Gökhan Tümgör, Didem Gülcü Taşkın, Buket Dalgıç, Gönül Çaltepe, Kaan Demirören, Güzide Doğan, Ceyda Tuna Kırsaçlıoğlu, Duran Arslan, İshak Abdurrahman Işık, Hülya Demir, Özlem Bekem, Yasin Şahin, Nevzat Aykut Bayrak, Mukadder Ayşe Selimoğlu, Sibel Yavuz, İbrahim Ethem Taşkaya, Derya Altay, the VICTORIA Study Group, Didem Gülcü Taşkın

**Affiliations:** ^1^ Department of Pediatrics, Division of Pediatric Gastroenterology, Ankara University School of Medicine, Ankara, Türkiye; ^2^ Department of Pediatric Gastroenterology, Çukurova University School of Medicine, Adana, Türkiye; ^3^ Department of Pediatrics, Adana City Training and Research Hospital, Adana, Türkiye; ^4^ Department of Pediatric Gastroenterology, Gazi University School of Medicine, Ankara, Türkiye; ^5^ Department of Pediatric Gastroenterology, Ondokuz Mayıs University School of Medicine, Samsun, Türkiye; ^6^ Department of Pediatrics, Yüksek İhtisas Training and Research Hospital, Bursa, Türkiye; ^7^ Department of Pediatrics, Haseki Training and Research Hospital, İstanbul, Bezmialem Vakıf University, İstanbul, Türkiye; ^8^ Department of Pediatric Gastroenterology, Erciyes University School of Medicine, Kayseri, Türkiye; ^9^ Department of Pediatrics, University of Health Sciences Antalya Training and Research Hospital, Antalya, Türkiye; ^10^ Department of Pediatric Gastroenterology, Hacettepe University School of Medicine, Ankara, Türkiye; ^11^ University of Health Sciences, Dr. Behçet Uz Children's Hospital, İzmir, Türkiye; ^12^ Department of Pediatrics, Mersin City Training and Research Hospital, Mersin, Türkiye; ^13^ University of Health Sciences, Zeynep Kamil Women and Children's Training and Research Hospital, İstanbul, Türkiye; ^14^ Department of Pediatric Gastroenterology, İnönü University School of Medicine, Malatya, Türkiye

**Keywords:** neuromuscular disease, hypertransaminasemia, elevated transaminase, Duchenne muscular dystrophy, Becker muscular dystrophy, Pompe disease

## Abstract

**Introduction:**

Elevated transaminases and/or creatine phosphokinase can indicate underlying muscle disease. Therefore, this study aims to determine the frequency of Duchenne muscular dystrophy/Becker muscular dystrophy (DMD/BMD) in male children and Pompe disease (PD) in male and female children with isolated hypertransaminasemia.

**Methods:**

This multi-center, prospective study enrolled patients aged 3–216 months with serum alanine transaminase (ALT) and/or aspartate transaminase (AST) levels >2× the upper limit of normal (ULN) for ≥3 months. Patients with a known history of liver or muscle disease or physical examination findings suggestive of liver disease were excluded. Patients were screened for creatinine phosphokinase (CPK) levels, and molecular genetic tests for DMD/BMD in male patients and enzyme analysis for PD in male and female patients with elevated CPK levels were performed. Genetic analyses confirmed PD. Demographic, clinical, and laboratory characteristics of the patients were analyzed.

**Results:**

Overall, 589 patients [66.8% male, mean age of 63.4 months (standard deviation: 60.5)] were included. In total, 251 patients (188 male and 63 female) had CPK levels above the ULN. Of the patients assessed, 47% (85/182) of male patients were diagnosed with DMD/BMD and 1% (3/228) of male and female patients were diagnosed with PD. The median ALT, AST, and CPK levels were statistically significantly higher, and the questioned neurological symptoms and previously unnoticed examination findings were more common in DMD/BMD patients than those without DMD/BMD or PD (*p* < 0.001).

**Discussion:**

Questioning neurological symptoms, conducting a complete physical examination, and testing for CPK levels in patients with isolated hypertransaminasemia will prevent costly and time-consuming investigations for liver diseases and will lead to the diagnosis of occult neuromuscular diseases.

**Trial Registration:**

Clinicaltrials.gov NCT04120168.

## Introduction

Elevated levels of transaminases [serum alanine transaminase (ALT) and aspartate transaminase (AST)] can indicate underlying acute or chronic liver diseases or can be the result of medical drug treatments (1–3). However, while the majority of cases are attributed to these causes, cases of asymptomatic hypertransaminasemia have been reported in patients with an underlying muscle disease (4–7).

In the first instance, it is routine in clinical practice for children with elevated transaminase to undergo various laboratory tests and invasive techniques such as a liver biopsy to rule out the diagnosis of chronic liver disease. This is done before considering a potential diagnosis of muscle diseases such as Duchenne muscular dystrophy/Becker muscular dystrophy (DMD/BMD) (4, 5, 7, 8).

The process of obtaining a confirmatory diagnosis for DMD/BMD is often long and complex and does not commence until symptoms have become apparent. An earlier diagnosis at the pre-symptomatic stage will allow earlier access to treatment before the clinical progression of muscle involvement and provide an opportunity for genetic counseling.

In addition to the elevated ALT and/or AST levels, the elevation of creatine phosphokinase (CPK), a specific enzyme of the muscle tissue, indicates muscle damage or muscle disorders. Testing for CPK levels in a patient with elevated ALT and/or AST levels may help prevent the historic unnecessary, invasive, and costly diagnostic laboratory tests and procedures for the etiology of liver disease.

This study aims to determine the frequency of DMD/BMD in male pediatric subjects and Pompe disease in male and female pediatric subjects with isolated transaminase elevation ≥3 months. Prior to the initiation of this study, a literature review indicated that no publications investigated the frequency of muscular diseases in children with isolated transaminase elevation, apart from some case reports (4–7).

## Methods

The study was a multi-center, prospective, non-drug screening study conducted at multiple centers across Türkiye. Routine care of the patients was maintained during the study.

### Study population

The study included male and female patients aged 3–216 months, with serum transaminase levels (serum ALT and/or AST levels) >2 of the upper limit of normal (ULN) for at least 3 months, who were willing to sign and/or have a legal representative sign the written consent form. Patients who were younger than 3 months or older than 216 months; had a known history of liver disease, muscle disease, or rheumatologic disease; had a clinical history or physical examination findings that supported the possibility of liver disease (such as jaundice, variceal bleeding, hepatomegaly, splenomegaly, and ascites); were in the intensive care unit; or had known congenital anomalies, organ failure, or elevated levels of serum gamma-glutamyl transferase and total or direct bilirubin were excluded. In our study, obesity was not an exclusion criterion because obesity may be present in muscular dystrophies (9).

### Study design

During the screening process, demographic data, medical histories regarding the age of onset of crawling and walking, neurological symptoms such as fatigue with walking and exercise, gait disturbances (walking on tiptoes, waddling gait, and difficulty in climbing stairs, running, and jumping), pain and weakness in leg and hip muscles and frequent falls, and family histories of the patients included in the study were collected. Physical examination information such as presence of pseudohypertrophy, hyperlordosis, kyphosis, scoliosis, hypotony and Gower's sign was recorded. History of evaluation for elevated transaminases related with a liver disease already performed such as for Hepatitis B virus (HBV) and Hepatitis C virus (HCV) infection, autoimmune hepatitis, Wilson's disease, alpha-1 antitrypsin deficiency, Celiac disease and metabolic diseases, and whether abdominal ultrasonography and liver biopsy was performed were recorded. The presenting reason for evaluating ALT/AST levels and the first detection time of ALT/AST elevation were documented. The last ALT and AST levels were recorded, and a sample to assess the CPK level was sent to the laboratory and recorded afterward. After the screening evaluations, enzyme analysis for Pompe disease in girls and boys with elevated CPK levels and molecular genetic tests for DMD/BMD in boys with elevated CPK levels were performed.

For the detection of dystrophin gene duplication and deletion in cases of DMD/BMD, multiplex ligation-dependent probe amplification (MLPA) was employed. In patients without duplication or deletion identified through MLPA, re-screening for other mutations was conducted using gene sequencing. These analyses were performed using the dried blood spot method by CENTOGENE AG Laboratories in Germany.

To diagnose Pompe disease, Duzen Laboratories in Türkiye performed the acid alpha-glucosidase enzyme (GAA) test and gene sequencing test in the same samples with low enzyme activity by obtaining an estimated amount of 10 mL venous/capillary blood.

The study population is classified into the total group, which included all patients who participated in the study; group 1, which included all patients not diagnosed with DMD/BMD or Pompe disease; group 2, which included patients diagnosed with DMD/BMD; and group 3, which included patients diagnosed with Pompe disease.

### Study endpoints

The endpoints of the study include determining the frequency of DMD/BMD in boys and Pompe disease in girls and boys with isolated transaminase elevation for at least 3 months and determining the demographic and clinical characteristics of these patients.

### Statistical analysis

Demographic characteristics, disease history, clinical data, and evaluation criteria data were summarized using descriptive statistics. Parametric or non-parametric tests were used as appropriate for subgroup analyses. Continuous variables are presented as mean, median, standard deviation (SD), maximum, and number of unmissed observations. Categorical data are presented as absolute and relative frequency (including the category named “missing” in suitable situations) for each category. The level of statistical significance was determined as *p* < 0.05.

### Consent and ethics procedures

Before enrollment, each patient or legal guardian had to provide written informed consent. This study complied with the guidelines of Good Clinical Practice and the International Conference on Harmonization, local rules and obligations, and the World Medical Association Helsinki Declaration.

The protocol, patient information page, and informed consent form were presented to the relevant Ethics Committee, and the study was registered to ClinicalTrials.gov (NCT04120168).

## Results

### Patient disposition and baseline characteristics

**Figure 1:**
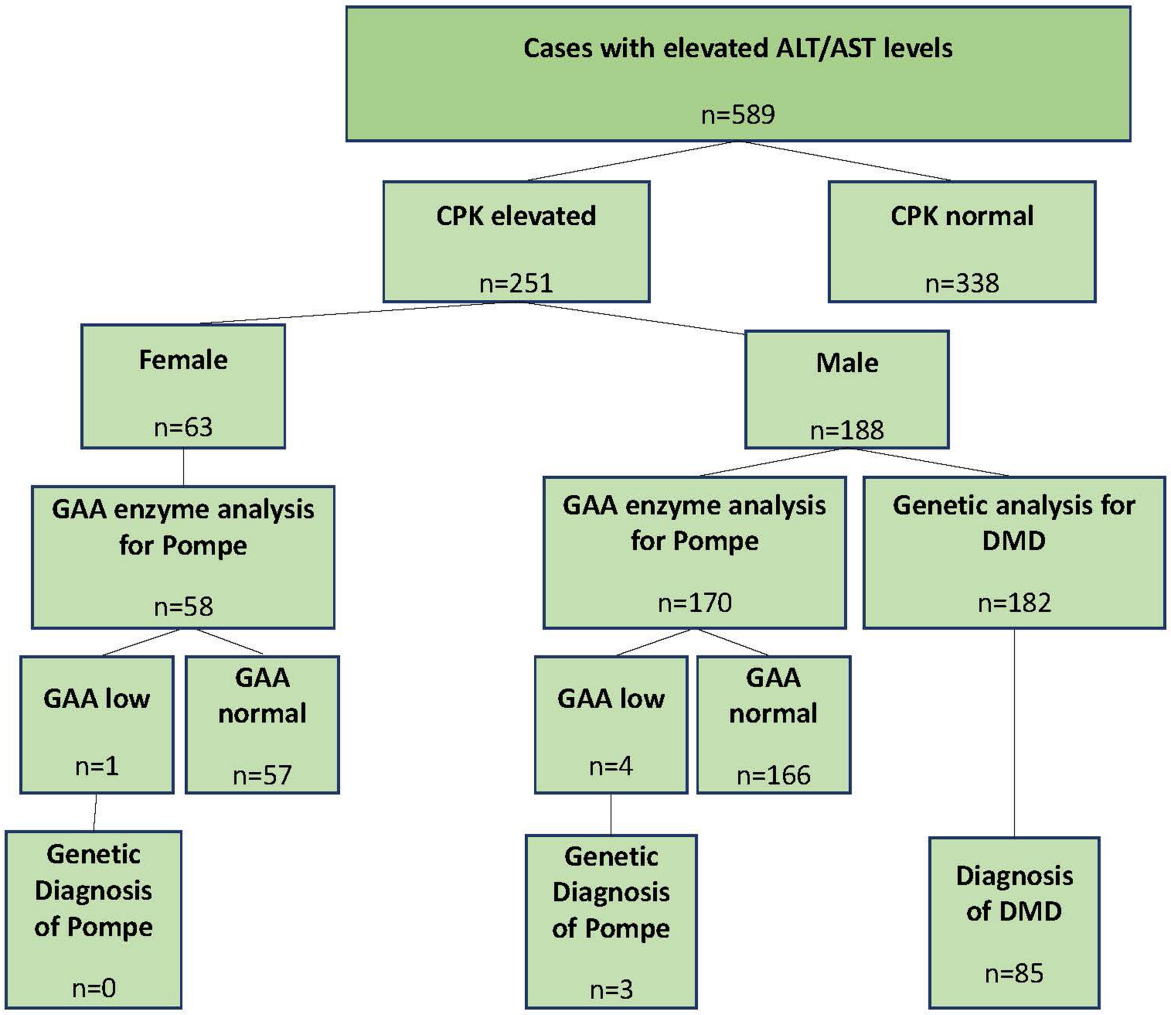
Study disposition. ALT, alanine aminotransferase; AST, aspartate aminotransferase; CPK, creatinine phosphokinase; DMD, Duchenne muscular dystrophy; GAA, acid alpha glucosidase.

The study included 589 patients from 45 centers between April 2019 and January 2022 (primary completion date, Figure 1). Of these patients, 66.8% were male and 33.2% were female, with a mean age of 63.4 months (SD: 60.5). The median age (range) was 39 months (3–216 months). The ALT and/or AST levels [overall mean (SD): ALT 196.15 (215.79) U/L; AST 180.70 (368.79) U/L] were 4.9 (5.7) and 4.5 (10.4) times above the ULN. The CPK values were recorded for all cases, and 251 patients [188 male (74.9%) and 63 female (25.1%)] had CPK values above the ULN. The mean age of these patients was 61.8 (59.1) months.

In groups 1 and 2, the most common reasons for measuring ALT/AST levels were related to diagnosing any disease and routine monitoring. The distribution of the groups was not statistically significant (test = 0.902, *p* = 0.342) (Table 1).

**Table 1:** Reasons for evaluating ALT/AST elevation.

	Group 1		Group 2		Group 3	
	Frequency	Percentage	Frequency	Percentage	Frequency	Percentage
Previously elevated	110	21.96	13	15.48	1	33.33
Prior to starting on an enzyme-level-increasing drug	9	1.80	0	0.00	0	0.00
Due to any disease	202	40.32	33	39.29	2	66.67
Control before surgical intervention	25	4.99	9	10.71	0	0.00
Routine control	147	29.34	22	26.19	0	0.00
Other	8	1.60	7	8.33	0	0.00

ALT, alanine aminotransferase; AST, aspartate aminotransferase.

Of the 182 male patients, 85 were diagnosed with DMD/BMD (group 2), and of the 228 male and female patients, three were diagnosed with Pompe disease (group 3) by genetic testing. The remaining 501 patients comprised group 1. The mean (SD) and median (range) ages were 66.0 (62.9), 40 (3–216); 45.1 (37.5), 34 (3–197); and 78.3 (115.1), 18 (6–211) in groups 1, 2, and 3, respectively. The age distributions of the groups were not statistically significant (*p* = 0.431).

The overall mean (SD) CPK level was 2,530.48 (6,205.75) U/L, which was 14.6 (37.1) times above the ULN. The median ALT, AST, and CPK levels were 124, 100, and 135 U/L in group 1; 221, 199, and 9,158 U/L in group 2; and 80, 219, and 1,101 U/L in group 3 (Table 2). The median ALT, AST, and CPK levels of group 2 were statistically significantly higher than those of group 1 (*p* < 0.001). Statistical analysis was not performed for group 3 as the sample number was insufficient.

**Table 2:** ALT, AST, and CPK levels and multiples of ULN.

		Group 1	Group 2	Group 3
AST U/L	Mean (SD)	170.32 (395.38)	240.16 (137.32)	207.00 (73.74)
	Median (min, max)	100.50 (33.00, 8,133.00)	199.00 (57.00, 745.00)	219.00 (128.00 274.00)
AST times above ULN	Mean (SD)	4.26 (11.19)	5.60 (2.50)	4.73 (1.33)
	Median (min, max)	2.50 (0.70, 232.40)	4.50 (1.40, 19.10)	5.50 (3.20, 5.50)
ALT U/L	Mean (SD)	187.01 (221.71)	251.64 (172.12)	128.67 (86.90)
	Median (min, max)	124.00 (11.00, 2,427.00)	221.00 (29.00, 892.00)	80.00 (77.00, 229.00)
ALT times above ULN	Mean (SD)	4.76 (5.97)	5.86 (4.12)	2.63 (1.38)
	Median (min, max)	3.10 (0.30, 69.30)	4.80 (0.60, 22.30)	2.10 (1.60, 4.20)
CPK U/L	Mean (SD)	1,069.88 (4,332.62)	11,070.69 (8,352.59)	1,069.67 (540.68)
	Median (min, max)	135.50 (12.00, 69,570.00)	9,158.00 (300.00, 40,000.00)	1,101.00 (514.00, 1,594.00)
CPK times above ULN	Mean (SD)	7.17 (29.31)	58.31 (47.37)	5.17 (2.71)
	Median (min, max)	0.7 (0.1, 406.8)	49.2 (3.0, 204.5)	4.9 (2.6, 8.0)

ALT, alanine aminotransferase; AST, aspartate aminotransferase; CPK, creatinine phosphokinase; SD, standard deviation; ULN, upper limit of normal.

The mean (SD) (months) of the first detection of ALT/AST elevation was 11.4 (16.3) months overall and 11.6 (16.4), 10.4 (15.7), and 15.7 (20.2) months in groups 1, 2, and 3, respectively. The time of the first detection of ALT/AST elevation distributions of the groups was not statistically significant (*p* = 0.543). The maximum time (months) of the first detection of ALT/AST elevation was 120, 90, and 39 months in groups 1, 2, and 3, respectively.

The most common evaluations performed to determine the causes of ALT/AST elevation were similar in groups 1 and 2, respectively: HBV (89.8% and 84.7%), HCV (88.6% and 81.2%), and alpha-1 antitrypsin deficiency (76.1% and 63.5%). Abdominal ultrasonography was performed in 90.1% and 80% and liver biopsy in 12% and 1.2% in groups 1 and 2, respectively (Table 3).

**Table 3:** Evaluations performed to find out the causes leading to ALT/AST elevation.

	Group 1		Group 2		Group 3	
	Frequency	Percentage	Frequency	Percentage	Frequency	Percentage
Evaluation for HBV	450	89.82	72	84.71	2	66.67
Evaluation for HCV	444	88.62	69	81.18	2	66.67
Evaluation for autoimmune hepatitis	331	66.07	43	50.59	2	66.67
Evaluation for Wilson disease	307	61.28	48	56.47	1	33.33
Evaluation for alpha-1 antitrypsin deficiency	381	76.05	54	63.53	2	66.67
Evaluation for celiac disease	328	65.47	49	57.65	1	33.33
Evaluation for metabolic diseases	326	65.07	42	49.41	1	33.33
Ultrasonography	451	90.02	68	80.00	3	100.00
Liver biopsy	60	11.98	1	1.18	0	0.00

HBV, hepatitis B virus; HCV, hepatitis C virus.

### Clinical characteristics

Table 4 provides the mean age of onset of crawling and walking for each group. Data for a number of patients were missing due to the inability to remember or patients not yet crawling or walking. Significant differences in the ages of onset of crawling and walking were demonstrated between groups 1 and 2 (test = 3.029, *p* = 0.003 and test = 2.605, *p* = 0.009, respectively).

**Table 4:** Age of starting to crawl or walk.

Crawling (months)	Group 1	Group 2	Group 3	Total group
*n*	274	59	1	334
Mean	8.68	9.66	11.00	8.86
Standard deviation	2.04	3.34	–	2.35
Median	9.00	9.00	11.00	9.00
Minimum	25.00	24.00	11.00	25.00
Maximum	4.00	6.00	11.00	4.00
Walking (months)	Group 1	Group 2	Group 3	Total group
*n*	281	60	2	343
Mean	13.26	14.87	12.50	13.54
Standard deviation	4.08	4.39	0.71	4.16
Median	12.00	14.00	12.50	12.00
Minimum	48.00	36.00	13.00	48.00
Maximum	8.00	9.00	12.00	8.00

**Figure 2:**
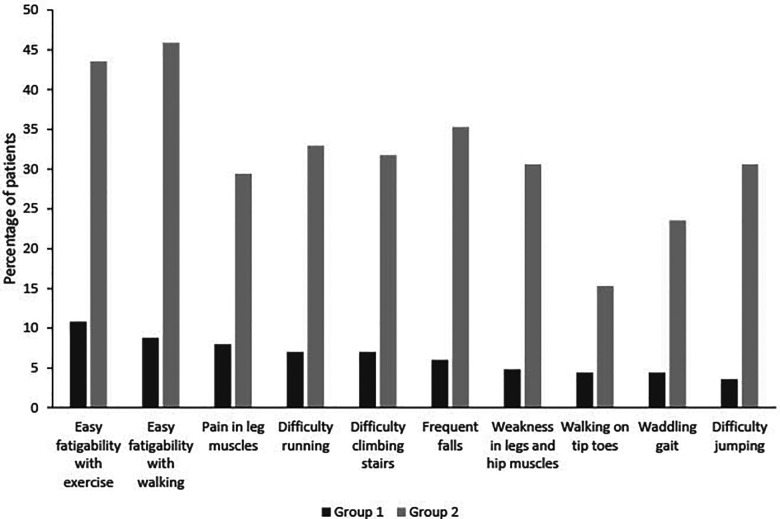
Evaluation of symptoms in groups 1 and 2.

In group 1 overall, 10.8% of the patients had easy fatigability with exercise, 8.8% had easy fatigability with walking, and 8.0% had pain in leg muscles. In group 2, 45.9% had easy fatigability with walking, 43.5% had easy fatigability with exercise, and 35.3% had frequent falls (leading neurologic symptoms). A comparison between groups 1 and 2 for the presence of each symptom demonstrated that patients in group 2 were statistically more likely to have those symptoms than those in group 1 (*p* < 0.001, Figure 2).

In patients older than 2 years, in group 1, 17.5% of patients reported easy fatigability with exercise, 14.5% reported easy fatigability with walking, and 13.1% reported pain in leg muscles (Table 5). In group 2, the percentage of patients reporting symptoms increased, with 59.3% reporting easy fatigability with walking, 57.6% reporting easy fatigability with exercise, and 40.7% reporting difficulty running. A comparison between groups 1 and 2 for the presence of each symptom demonstrated that patients in group 2 were statistically more likely to have those symptoms than those in group 1 (*p* < 0.001).

**Table 5:** Neurologic symptoms and physical findings in patients aged ≥2 years.

	Group 1 (*n* = 296)		Group 2 (*n* = 56)		Total group (*n* = 355)	
	Frequency	Percentage	Frequency	Percentage	Frequency	Percentage
Neurological symptoms						
Easy fatigability with walking	43	14.48	35	59.32	78	21.91
Walking on tiptoes	17	5.72	10	16.95	27	7.58
Staggering gait	21	7.07	15	25.42	36	10.11
Easy fatigability with exercise	52	17.51	34	57.63	86	24.16
Pain in leg muscles	39	13.13	23	38.98	62	17.42
Difficulty climbing stairs	32	10.77	23	38.98	55	15.45
Difficulty running	33	11.11	24	40.68	57	16.01
Difficulty jumping	16	5.39	22	37.29	38	10.67
Frequent falls	21	7.07	20	33.90	41	11.52
Weakness in legs and hip muscles	21	7.07	19	32.20	40	11.24
Physical findings						
Gower's sign	7	2.36	16	28.57	23	6.48
Pseudohypertrophy	13	4.39	25	44.64	38	10.70
Hyperlordosis	5	1.69	7	12.50	12	3.38
Kyphosis	5	1.69	0	0.00	5	1.41
Scoliosis	9	3.04	2	3.57	11	3.10
Hypotony	4	1.35	1	1.79	5	1.41

A statistically significant difference in inquiries regarding neurological symptoms was obtained for frequent falls [mean (SD) age: with 50.7 (25.0) vs. without 69.1 (40.6), *p* = 0.046]; statistically significant differences for the other symptoms were not noted (Table 6).

**Table 6:** Comparison of ages regarding inquiries for neurologic symptoms.

	Inquiry result	Mean age (months)	SD	Median	Min.	Max.	Test	*p*
Easy fatigability with walking	Yes	62.68	30.37	56.00	24.00	141.00	0.067	0.946
	No	61.60	46.99	40.50	26.00	197.00		
Walking on tiptoes	Yes	53.30	18.33	53.50	25.00	82.00	1.318	0.199
	No	64.73	39.55	54.50	26.00	197.00		
Staggering gait	Yes	63.74	40.15	55.00	25.00	197.00	0.434	0.667
	No	59.67	26.58	51.00	34.00	127.00		
Easy fatigability with exercise	Yes	63.22	30.65	56.00	25.00	141.00	0.156	0.877
	No	61.73	44.82	42.00	26.00	197.00		
Pain in leg muscles	Yes	69.22	33.66	61.00	27.00	141.00	0.962	0.341
	No	59.48	39.26	48.00	26.00	197.00		
Difficulty climbing stairs	Yes	58.82	32.39	49.50	25.00	141.00	0.650	0.518
	No	65.22	39.66	55.50	26.00	197.00		
Difficulty running	Yes	68.61	34.01	61.00	25.00	141.00	1.055	0.296
	No	58.16	38.49	48.00	26.00	197.00		
Difficulty jumping	Yes	62.67	33.19	56.00	25.00	141.00	0.009	0.993
	No	62.58	39.26	54.00	26.00	197.00		
Frequent falls	Yes	50.74	24.95	44.00	25.00	127.00	2.050	0.046
	No	69.06	40.58	59.00	26.00	197.00		
Weakness in legs and hip muscles	Yes	58.61	26.67	55.00	27.00	127.00	0.646	0.521
	No	64.61	40.99	54.50	25.00	197.00		

SD, standard deviation.

With respect to overall physical examination findings, in group 1, pseudohypertrophy was seen in 2.6% of patients, Gower's sign in 1.4%, and hyperlordosis in 1.2%. In group 2, pseudohypertrophy was seen in 37.6% of patients, Gower's sign in 22.3%, and hyperlordosis in 8.2%. The likelihood of experiencing these physical examination findings was significantly higher in group 2 compared with group 1 (*p* < 0.001).

In patients older than 2 years, in Group 1, pseudohypertrophy was seen in 4.4%, Gower's sign in 2.4%, and hyperlordosis in 1.7% of cases (Table 5). In Group 2, pseudohypertrophy was seen in 44.6%, Gower's sign in 28.6%, and hyperlordosis in 12.5% of cases. These physical signs were significantly higher in group 2 compared with group 1 (*p* < 0.001). An analysis of the ages regarding the presence or absence of physical examination findings in cases aged above 2 years in group 2 revealed no statistically significant differences in any of the findings (Table 7).

**Table 7:** Physical examination findings in patients ≥2 years in group 2.

Physical examination finding	Result of finding	Mean (months)	SD	Median	Min.	Max.	Test	*p*
Gower's sign	No	54.50	26.91	49.50	25.00	127.00	1.234	0.224
	Yes	66.03	39.93	56.00	27.00	197.00		
Pseudohypertrophy	No	55.71	22.53	48.00	30.00	127.00	1.329	0.190
	Yes	68.13	44.57	55.50	25.00	197.00		
Hyperlordosis	No	56.29	32.54	48.00	34.00	127.00	0.540	0.602
	Yes	63.55	37.49	55.00	25.00	197.00		
Kyphosis	No	–	–	–	–	–		
	Yes	62.61	36.69	54.50	25.00	197.00		
Scoliosis	No	91.50	50.20	91.50	56.00	127.00		
	Yes	61.50	36.27	52.50	25.00	197.00		
Hypotony	No	–	–	–	–	–		
	Yes	63.62	37.02	55.50	25.00	197.00		

When a sufficient number of observations was not obtained in the groups, descriptive statistics and significance test results for comparisons could not be achieved.

SD, standard deviation.

### Genetic analysis

Of the 182 male patients who underwent genetic testing for the diagnosis of DMD/BMD, 85 were diagnosed with DMD. The most common mutations were exon 45–47 deletion (*n* = 7) and exon 45–48 deletion (*n* = 4). Supplementary Table S1 shows the full range of genetic mutations.

Of the 228 patients who underwent enzymatic testing for the diagnosis of Pompe disease (170 males and 58 females), five patients had low enzyme levels (4 males and 1 female). Three of these patients were subsequently confirmed to have Pompe disease (three males) through genetic testing. The genetic mutations identified for these patients were c.-32–13T>G, c.1655T>C, and c.258dupC. Details regarding the demographic characteristics, medical history, neurological symptoms and findings, GAA levels, and genetic mutations for these patients are available in Table 8.

**Table 8:** Baseline and clinical characteristics of patients with confirmed Pompe disease.

Sex	Age (year/month)	Consanguinity	Age of crawling	Age of walking	Positive neurological sign	Positive physical examination findings	Time of first detection of ALT/AST elevation (months ago)	ALT, AST, and CPK values and multiples of ULN	GAA enzyme value	Mutation	Variant type	ACMG classification
Male	17 years and 7 months/211 months	No	Not remembered	12 months	None	None	39	AST: 128 (3.2-fold) ALT: 80 (2-fold) CPK: 1,594 (8-fold)	10.8	c.-32–13T>G	Control region variant	Pathogenic
Male	1 year and 6 months/18 months	Yes	11 months	13 months	Waddling gait, frequent falls	None	5	AST: 274 (5.5-fold) ALT: 229 (4.2-fold) CPK: 1,101 (4.9-fold)	12.7	c.1655T>C	Missense variant	Pathogenic
Male	0 years and 6 months/6 months	No	Not started crawling yet	Not started walking yet	None	Hypotony	3	AST: 219 (5.5-fold) ALT: 77 (1.6-fold) CPK: 514 (2.6-fold)	2.6	c.258dupC	Frameshift variant	Pathogenic

ACMG, American College of Medical Genetics; ALT, alanine aminotransferase; AST, aspartate aminotransferase; CPK, creatinine phosphokinase; GAA, acid alpha glucosidase; SD, standard deviation; ULN, upper limit of normal.

## Discussion

The results described here are the first, to the best of the authors knowledge, from a dedicated large study in pediatric patients with isolated transaminase elevation. These findings align with previous case reports (4, 5, 7). A retrospective review of 232 Chinese patients noted that more than 97% of patients diagnosed with DMD or BMD initially presented with elevated transaminases (10).

Of the patients with elevated CPK levels above the ULN who were assessed, 47% (85/182) of male patients were found positive for DMD/BMD, and 1% (3/228) of male and female patients tested positive for Pompe disease. It is important to note that the patients included in this study did not initially present with symptoms associated with DMD/BMD or Pompe disease such as easy fatigability with walking and exercise, gait disturbances (walking on tiptoes, waddling gait, or difficulty in climbing stairs, running, or jumping), pain or weakness in leg or hip muscles, or frequent falls. These symptoms were only discovered during the screening process of this study, which shows the importance of a gathering a detailed history regarding neurological symptoms in evaluating a patient with isolated transaminase elevation.

In a systemic physical examination, it is essential to perform neurological examinations as a key component of clinical investigations for patients with isolated hypertransaminasemia. From our study, we note that a larger proportion of patients in group 2 (DMD/BMD diagnosis group) exhibited occult or minimally symptomatic neurological signs such as Gower's sign and pseudohypertrophy than those in group 1 (patients with no DMD/BMD or Pompe diagnosis), which were not reported prior to the inclusion of the patients in this study. This proportion increased in patients aged over 2 years. Although the frequency of occult muscle diseases is well known among pediatricians, our results highlight the frequency of hepatic-driven myopathies, which should be as equally well known.

Not recognizing that a muscle disease could be the cause of transaminase elevation and failing to detect some occult neurological symptoms or signs due to a lack of structured inquiry or examination could cause a delay in making an accurate diagnosis. In our study, the median time to diagnose DMD/BMD or Pompe disease after detecting transaminase elevation was around a year. In some patients, the delay was considerable, even reaching 7 years.

As described earlier, DMD and BMD are progressive muscle dystrophies caused by mutations in the DMD gene, affecting muscle development and causing muscle weakening and, ultimately, cardiac and respiratory issues (11–13). DMD is the most prevalent among muscle dystrophies, affecting approximately 7.1 cases (95% CI: 5.0–10.1) per 100,000 males and 2.8 cases (95% CI: 1.6–4.6) per 100,000 in the general population, while the pooled global DMD birth prevalence rate is 19.8 (95% CI: 16.6–23.6) per 100,000 live male births (14). Symptoms of the disease manifest in the form of fatigue and skeletal muscle weakness and trouble performing activities such as getting up from a lying position, going uphill, and climbing the stairs, which progressively start in early childhood and eventually lead to a loss of ambulation. In BMD, weakness sets in later, and similar signs are first observed at school-age years. The disease course is mild and protracted, allowing patients to remain ambulatory until late adolescence or young adulthood (11–13).

Pompe disease is caused by mutations in the GAA gene, leading to the accumulation of glycogen and affecting skeletal and cardiac muscles, and can appear any time from infancy, infantile-onset Pompe disease (IOPD), to adulthood, late-onset Pompe disease (LOPD), with an overall prevalence rate of between 1 in 40,000 and 1 in 351,000 (15–17). Cardiac involvement is more severe and fatal in the first year of life in IOPD, while skeletal muscle symptoms manifest in teenage years or even later. On the other hand, LOPD has a slow progression rate. The first sign is usually weakness of the legs and hips, which leads to a waddling gait. Patients often have a history of muscle pain and frequent falling. Lordosis, kyphosis, and scoliosis may be seen at later ages. IOPD is more easily recognized due to its specific symptoms; however, the disease may be hard to diagnose in older children and adults. Gradually appearing signs may not be recognized and may be confused with other neuromuscular disorders with similar signs, which causes long diagnostic delays.

A diagnostic evaluation of a child with isolated, asymptomatic transaminase elevation should include another simple laboratory test for CPK. CPK levels are a sensitive biochemical marker for the early detection of myopathies and are a potential marker for undiagnosed muscle diseases; however, this concept is not yet widely accepted or integrated into clinical practice. Testing for this marker can eliminate costly and time-consuming investigations for a number of liver diseases. In our study, most of the laboratory tests for liver diseases and abdominal ultrasonography were already performed before detecting CPK elevations. CPK testing was performed in these patients for the first time during this study, which caused delays in eventual diagnosis. Since liver biopsy was not performed in all patients with obesity, we cannot exclude steatotic liver disease as a potential undiagnosed condition within our study group. An earlier case report has illustrated that a link between DMD/BMD and non-alcoholic fatty liver disease does exist (18).

An early diagnosis of DMD/BMD and Pompe disease will provide the patients with access to supportive therapy not only for ambulation but also for developing comorbidities such as cardiac and respiratory involvement. In addition, research into these diseases is continually evolving, with the latest treatment options such as genetic therapy targeted specifically at the type of mutation. Therefore, one of the keys to successful treatment is the early identification of affected patients in the disease course to ensure timely intervention and attenuate symptom development (19).

A limitation of this study should be noted. For patients with CPK elevation, further investigation regarding the cause of a muscular disorder other than that for DMD/BMD and Pompe disease was not performed. Additional investigation in these patients might have been warranted to investigate the cause of the CPK elevation.

## Conclusions

This study confirms previous reports of the importance of considering the link between isolated elevated transaminases and diagnoses of muscle diseases such as DMD/BMD and Pompe disease and may provide primary care physicians and pediatricians with valuable insights for improving their day-to-day clinical practice. Patients presenting with isolated/asymptomatic hypertransaminasemia should be evaluated in more detail for the possibility of a neuromuscular disease. In addition to pediatricians, pediatric gastroenterologists should be aware of patients with transaminase elevation who are commonly referred to them for the evaluation of a supposedly silent hepatic disease and should consider the possibility of an underlying muscular disorder. Targeting specific history-taking, such as questioning neurological symptoms, performing basic neurological physical examination, and introducing routine testing of CPK levels, assists in the diagnostic process for specific muscular diseases, supporting earlier initiation of treatment and preventing lengthy diagnostic delays. CPK testing should always be included in the first step of laboratory investigation for isolated hypertransaminasemia, even before the onset of any symptom of a muscular disorder.

## Data Availability

The original contributions presented in the study are included in the article/**Supplementary Material**, further inquiries can be directed to the corresponding author.

## VICTORIA Study Group

Adana City Training and Research Hospital, Adana, Türkiye (**Didem Gülcü Taşkın**). Afyonkarahisar Health Sciences University, Faculty of Medicine, Department of Pediatric Gastroenterology, Afyonkarahisar, Türkiye (**Ayşegül Bükülmez**). Ankara City Hospital, Ankara, Türkiye (**Arzu Meltem Demir**). Ankara Training and Research Hospital, Ankara, Türkiye (**Yavuz Tokgöz**). Ankara University School of Medicine, Department of Pediatric Gastroenterology, Ankara, Türkiye (**Aydan Kansu, Zarife Kuloğlu, Ceyda Tuna Kırsaçlıoğlu**). Bakırköy Sadi Konuk Training and Research Hospital, İstanbul, Türkiye (**Hasret Ayyıldız**). Başakşehir Çam Sakura Hospital, İstanbul, Türkiye (**Günsel Kutluk, Meryem Keçeli Başaran**). Başkent University School of Medicine, Department of Pediatric Gastroenterology, Ankara, Türkiye (**Oya Balcı Sezer**). Bursa Uludağ University School of Medicine, Department of Pediatric Gastroenterology, Bursa, Türkiye (**Tanju Başarır Özkan, Taner Özgür**). Bülent Ecevit University School of Medicine, Department of Pediatric Gastroenterology, Zonguldak, Türkiye (**Gonca Handan Üstündağ, Eda Somuncu**). Çukurova University School of Medicine, Department of Pediatric Gastroenterology, Adana, Türkiye (**Gökhan Tümgör, Sibel Yavuz, Ali İşlek**). Dr. Sami Ulus Children's Hospital, Ankara, Türkiye (**Ferda Özbay Hoşnut, Gülseren Evirgen Şahin**). Erciyes University School of Medicine, Department of Pediatric Gastroenterology, Kayseri, Türkiye (**Duran Arslan, Derya Altay**). Fırat University School of Medicine, Department of Pediatric Gastroenterology, Elazığ, Türkiye (**Yaşar Doğan, Uğur Deveci**). Gazi University School of Medicine, Department of Pediatric Gastroenterology, Ankara, Türkiye (**Buket Dalgıç, Kamercan Ceylan**). Gaziantep University Department of Pediatric Gastroentreology, Gaziantep, Türkiye (**Ahmet Baştürk**). Gülhane Training and Research Hospital, Department of Pediatric Gastroenterology, Ankara, Türkiye (**Necati Balamtekin, Melike Arslan**). Hacettepe University School of Medicine, Department of Pediatric Gastroenterology, Ankara, Türkiye (**Hülya Demir, Hayriye Hızarcıoğlu Gülşen**). Haseki Training and Research Hospital, İstanbul, Türkiye (**Güzide Doğan**, currently at: Bezmialem Vakıf University, Department of Pediatrics, İstanbul). Hitit University Department of Pediatrics, Çorum, Türkiye (**Atakan Comba**). İnönü University School of Medicine, Department of Pediatric Gastroenterology, Malatya, Türkiye (**Mukadder Ayşe Selimoğlu, İlknur Varol**). İstanbul Medeniyet University School of Medicine, Department of Pediatric Gastroenterology, İstanbul, Türkiye (**Sebahat Çam**). Karabük University School of Medicine, Department of Pediatrics, Karabük, Türkiye (**Eylem Sevinç, Erkan Doğan**). Karadeniz University School of Medicine, Department of Pediatrics, Trabzon, Türkiye (**Murat Çakır, Burcu Güven**). Keçiören Training and Research Hospital, Ankara, Türkiye (**Suna Selbuz**). Kırıkkale University School of Medicine, Department of Pediatric Gastroenterology, Kırıkkale, Türkiye (**Hacer Fulya Gülerman, Zeynep Arslan**). Kocaeli University School of Medicine, Department of Pediatric Gastroenterology, Kocaeli, Türkiye (**Ayşen Uncuoğlu**). Malatya Training and Research Hospital, Malatya, Türkiye (**Neslihan Gürcan Kaya**). Marmara University School of Medicine, Department of Pediatric Gastroenterology, İstanbul, Türkiye (**Deniz Ertem, Engin Tutar, Burcu Volkan**). Mersin City Training and Research Hospital, Mersin, Türkiye (**Yasin Şahin**). Mersin University School of Medicine, Department of Pediatric Gastroenterology, Mersin, Türkiye (**Yusuf Usta, Asuman Nur Karhan**). Ondokuz Mayıs University School of Medicine, Department of Pediatric Gastroenterology, Samsun, Türkiye (**Gönül Çaltepe, İbrahim Ethem Taşkaya**). Pamukkale University School of Medicine, Department of Pediatric Gastroenterology, Denizli, Türkiye (**Halil Kocamaz, Tuğba Gürsoy Koca**). Private Practise, Bursa, Türkiye (**Fatih Ünal**). Prof. Dr. Cemil Taşcıoğlu City Hospital, İstanbul, Türkiye (**Birol Öztürk, Cansu Altuntaş**). Selçuk University Faculty of Medicine, Department of Pediatric Gastroenterology, Konya, Türkiye (**Halil Haldun Emiroğlu, Meltem Gümüş**). Süleyman Demirel University School of Medicine, Department of Pediatric Gastroenterology, Isparta, Türkiye (**Mustafa Akçam**). Tepecik Training and Research Hospital, İzmir, Türkiye (**Yeliz Çağan Appak**, currently at İzmir Katip Celebi University, **Betül Aksoy**). Trabzon Kanuni Training and Research Hospital, Trabzon, Türkiye (**Elif Sağ**). University of Health Sciences Antalya Training and Research Hospital, Antalya, Türkiye (**İshak Abdurrahman Işık, Ulaş Emre Akbulut**). University of Health Sciences, Dr. Behçet Uz Children's Hospital, İzmir, Türkiye (**Özlem Bekem, Cahit Barış Erdur**). University of Health Sciences İstanbul Şişli Hamidiye Etfal Education and Research Hospital, Department of Pediatric Gastroenterology, Istanbul, Türkiye (**Nafiye Urgancı, Ayşe Merve Usta**). University of Health Sciences, Umraniye Training and Research Hospital, Pediatric Gastroenterology Clinic, İstanbul, Türkiye (**Coşkun Çeltik, Nelgin Gerenli**). University of Health Sciences, Zeynep Kamil Women and Children's Training and Research Hospital, İstanbul, Türkiye (**Nevzat Aykut Bayrak**). Yüksek İhtisas Training and Research Hospital, Bursa, Türkiye (**Kaan Demirören**).
