# Supplementary material for: The frequency of Duchenne muscular dystrophy/Becker muscular dystrophy and Pompe disease in children with isolated transaminase elevation: results from the observational VICTORIA study
Source: Front Pediatr. 2023 Sep 25;11:1272177. doi: 10.3389/fped.2023.1272177 (PMC10560724; doi:10.3389/fped.2023.1272177)
Supplement: Supplementary file 1 [file Table1.docx]

# Supplementary Material

**Supplementary Table 1. Genetic testing results for DMD/BMD patients (All were classified as pathogenic due to ACMG criteria)**

| **Mutation** | **Number of patients** |
| --- | --- |
| exon 45-47 deletion | 7 |
| exon 45-48 deletion | 4 |
| 45-47 hemizygote deletion | 3 |
| 48-51 hemizygote deletion | 3 |
| exon 45-55 deletion | 3 |
| 3-25 hemizygote deletion | 2 |
| 46-50 hemizygote deletion | 2 |
| 51-55 hemizygote deletion | 2 |
| exon 48 deletion | 2 |
| exon 48 hemizygote deletion | 2 |
| exon 48-49 deletion | 2 |
| exon 48-51 deletion | 2 |
| exon 51 hemizygote deletion | 2 |
| NM_004006.2:c.4069G>T (p.Glu1357*) (Non-sense) | 2 |
| 10-20 hemizygote deletion | 1 |
| 16-43 hemizygote deletion | 1 |
| 22-42 hemizygote deletion | 1 |
| 2-7 hemizygote duplication | 1 |
| 3-37 hemizygote deletion | 1 |
| 43-44 hemizygote duplication, 56-60 duplication | 1 |
| 45-47 deletion | 1 |
| 45-50 hemizygote deletion | 1 |
| 45-51 hemizygote deletion | 1 |
| 45-53 hemizygote deletion | 1 |
| 45-54 hemizygote deletion | 1 |
| 46-47 hemizygote deletion | 1 |
| 46-48 hemizygote deletion | 1 |
| 46-52 hemizygote deletion | 1 |
| 46-55 deletion | 1 |
| 48-49 deletion | 1 |
| 48-50 hemizygote deletion | 1 |
| 49-50 hemizygote deletion | 1 |
| 49-52 hemizygote deletion | 1 |
| 49-54 hemizygote deletion | 1 |
| 51-54 hemizygote deletion | 1 |
| 63-69 hemizygote deletion | 1 |
| 64-74 hemizygote duplication | 1 |
| 8-20 duplication | 1 |
| 8-9 hemizygote deletion | 1 |
| c.10108C>T (p.Arg3370*) (Non-sense) | 1 |
| c.1533_1536del | 1 |
| c.2869C>T | 1 |
| c.3288_3297dup | 1 |
| c.3455dup | 1 |
| exon 14-25 deletion | 1 |
| exon 2 | 1 |
| exon 3-13 | 1 |
| exon 39-42 | 1 |
| exon 45-49 deletion | 1 |
| exon 46-50 deletion | 1 |
| exon 46-52 deletion | 1 |
| exon 46-53 deletion | 1 |
| exon 48-52 deletion | 1 |
| exon 49-50 deletion | 1 |
| exon 52 hemizygote deletion | 1 |
| exon 5-51 deletion | 1 |
| NM_004006.2: p.(Met3285Asnfs*42) c.9854_9863del (Frameshift) | 1 |
| NM_004006.2:c.1812+1G>A (splice variant) | 1 |
| NM_004006.2:c.2381-2A>G (splice variant) | 1 |
| NM_004006.2:c.4519-2A>G (splice variant) | 1 |
| NM_004006.2:c.9560A>G (missense variant) | 1 |

ACMG, American College of Medical Genetics; BMD, Becker Muscular Dystrophy; DMD, Duchenne Muscular Dystrophy
